# Antiobesity effects of onion (*Allium cepa*) in subjects with obesity: Systematic review and meta‐analysis

**DOI:** 10.1002/fsn3.3426

**Published:** 2023-05-16

**Authors:** Min‐Yu Chung, Jin‐Taek Hwang, Soo‐Hyun Park

**Affiliations:** ^1^ Department of Food and Nutrition Gangseo University Seoul Korea; ^2^ Food Functionality Research Division Korea Food Research Institute Wanju Korea

**Keywords:** BMI, body fat, clinical trial, onion peel, triglyceride

## Abstract

Onions are rich in bioactive compounds and have been found to prevent various chronic diseases, including obesity. We performed a systematic review and meta‐analysis to investigate the antiobesity effect of onions. Studies were identified in PubMed/MEDLINE, Embase, Web of Science, and CENTRAL focusing on clinical trials evaluating the antiobesity effects of onion in obese subjects. The risk of bias in the studies was evaluated using Cochrane's Risk of Bias tool. The effect of onions was analyzed using data from the selected studies, and the results were indicated by weighted mean difference with 95% CI. The *I* ^2^ static test was used to examine heterogeneity between the studies. A total of 38 studies were reviewed, of which five clinical trials meeting the criteria were selected. As investigational products, onion peels were used in four studies and onions were used in one study. Following systematic review, it was determined that the risk of bias was generally low, and body weight, BMI, waist circumference, and triglyceride levels were significantly reduced in the onion groups compared to the placebo. In conclusion, onion intake had an antiobesity effect by reducing body weight and body fat, and this effect was particularly pronounced with onion peel.

## Graphical abstract

Forest plot for the effects of onions on BMI. Squares represent weighted mean differences derived from individual studies. Horizontal bars represent 95% CI. The Diamonds represent the overall combined mean difference.

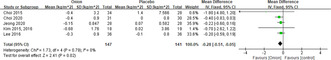

## 1 INTRODUCTION

According to the World Health Organization, obesity approximately tripled throughout the world from 1975 to 2016. Approximately 13% of the adult population (11% men, 15% women) are thought to be obese worldwide. Obesity has a negative impact on life expectancy, which is attributable to an increased incidence of obesity‐related pathological conditions including dyslipidemia, cardiovascular disorders, type 2 diabetes, and various cancers (Barone et al., 2016; Bastien et al., 2014; Franssen et al., 2011; Uzunlulu et al., 2016). Obesity can be prevented by regular physical activity, balanced nutrition, and the consumption of healthy foods. A number of studies have provided evidence for obesity‐preventing foods, which are mostly plant‐based and include garlic (Kagawa et al., 2020), ginger (Seo et al., 2021), tea (Zhou et al., 2022), and edible sprouts (Kim et al., 2022).

Onion (*Allium cepa*) has also been reported to support obesity prevention (Zhao et al., 2021). The biological properties of *Allium cepa* have been widely studied, which is likely due to the large number of phytonutrients it contains (Marrelli et al., 2018). The major phenolic compounds are quercetin, quercetin‐3‐glucoside, quercetin‐4‐glucoside, or rutin (Lee et al., 2017; Ren et al., 2017). In addition, various vitamins, minerals, sulfur‐based amino acids, phytosterols, and saponins are also known to contribute to the beneficial effects of onion on human health (Lanzotti, 2006; Marrelli et al., 2018).

The mechanisms of antiobesity effects have been proposed by a number of researchers. For instance, onion juice inhibits pancreatic lipase activity (IC50‐9.5 mg/mL) (Trisat et al., 2017), contributing to the inhibition of lipid absorption. Onion peel extract has also been shown to attenuate lipid accumulation in 3T3‐L1 cells by reducing lipogenesis‐related gene expression (Alshaker et al., 2015). The antiobesity effects of onion and its bioactive compounds have additionally been demonstrated in obese animal models. Yoshinari et al. (2012) observed decreased adipose tissue weight and serum lipids in diabetic Zucker rats fed with onion (*Allium cepa* Linn.) extract, while Kim et al. (2012) demonstrated that quercetin‐rich onion peel extract supplementation reduced mesenteric fat content as well as adipokine production in high‐fat diet‐fed obese rats.

Onion (including both bulb and peel) appears to be a promising natural agent for the prevention of obesity, with supportive in vitro and in vivo evidence. A number of clinical studies also support the antiobesity effect of onions in obese and overweight subjects (Choi et al., 2020; Jeong et al., 2020; Lee et al., 2016; Nishimura et al., 2019; Saghafi‐Asl & Ebrahimi‐Mameghani, 2014). The objective of the present study was to perform a meta‐analysis and systematic review of the antiobesity health benefits of onion in well‐designed placebo‐controlled clinical trials.

## 2 METHODS

### 2.1 Study registration

The study protocol (CRD42022304864) was registered in the PROSPERO database (https://www.crd.york.ac.uk/PROSPERO/).

### 2.2 Criteria for considering studies

The studies included in this systematic review and meta‐analysis were randomized controlled trials (RCTs) focusing on the antiobesity effect of onions. Studies evaluating the effect of the investigational product (IP) after single dose or onion supplementation as a part of another regimen were excluded from this review. The study subjects fit the criteria of overweight or obese.

### 2.3 Outcome measures

The antiobesity effect of onions was evaluated according to changes in body weight (BW), body mass index (BMI), body fat (BF), and waist circumference (WC). In addition, changes in blood lipid concentrations and obesity‐related hormones were also evaluated.

### 2.4 Search methods

The present study was conducted in accordance with the Preferred Reporting Items for Systematic Reviews and Meta‐analyses (PRISMA) guidelines. PubMed/Medline, Web of Science, EMBASE, and the Cochrane Central Register of Controlled Trials (CENTRAL) were chosen as the databases for literature searches and were reviewed for eligible studies published until May 2022. Publications were identified using the following search terms (also in combination with MESH and EMTREE terms): [(onion) OR (*Allium cepa*)] AND [(obes*) OR (overweight)] AND (placebo). To find additional studies, we also reviewed all references cited in the selected articles.

### 2.5 Selection of studies and data extraction

For the selection of eligible studies for inclusion, two reviewers independently screened the search results, with the first focusing on titles and abstracts and the second focusing on the full text. Any disagreements were resolved by consensus. Two reviewers independently extracted the following data using a standardized data extraction format, with inconsistent results resolved through confirmation and discussion of the material: authors, publication year, study design, baseline characteristics, sample size, supplementation, period, evaluation markers, and results.

### 2.6 Assessment of bias risk

Two reviewers independently assessed the risk of bias among the included studies using Cochrane's risk of bias tool, which assesses the risk of bias in studies according to random sequence generation, allocation concealment, blinding of the participants and personnel, blinding of outcome assessment, incomplete outcome data, selective reporting, and other biases.

### 2.7 Statistical analyses

A meta‐analysis was performed using Review Manager version 5.4 (Cochrane Collaboration, London, England). The units of all evaluation markers were properly standardized. We used the mean change and standard deviation (SD) values of the markers to investigate the effect size of the collected data. For studies that presented only standard error (SE), SE was converted into SD by multiplying by the square root of the sample size.

Pooled data were analyzed using a fixed‐effects model and the data were expressed as weighted mean difference (WMD) with 95% confidence interval (CI) for continuous outcomes. The *I* ^2^ statistic test was used to estimate the percentage of heterogeneity between studies; heterogeneity was confirmed if the *I* ^2^ value was 50% or more and if heterogeneity was confirmed, data were analyzed by applying a random‐effects model. Sensitivity analysis was also conducted using the leave‐one‐out method to estimate the effects of omission for each study (Maierean et al., 2017). *p*‐values of less than .05 were considered to be statistically significant.

## 3 RESULTS

### 3.1 Literature search

**FIGURE 1:**
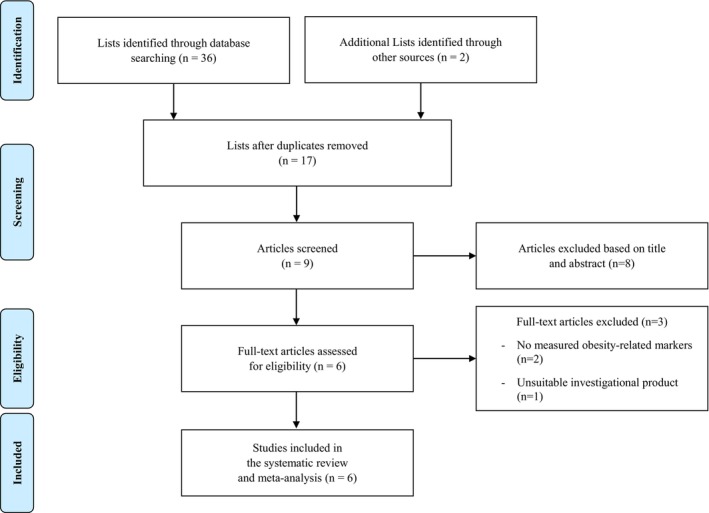
PRISMA flow diagram of the study selection process.

To evaluate the antiobesity effect of onions, we searched the literature publications using defined search terms in various databases. Among the 38 lists searched, 17 lists were selected by removing duplications before nine articles were eventually selected through title and abstract review. As a result, two articles that did not measure the anthropometric parameters and one article that evaluated the functionality of quercetin were additionally excluded through full‐text screening. Finally, six articles were selected for this systematic review and meta‐analysis (Figure 1).

### 3.2 Study description

The baseline characteristics of participants in the studies are detailed in Table 1. In our overall study review, two studies by Kim & Yim (2015, 2016) were judged to be from the same trial, so a total of five study results were collected by integrating the two study reports into one. All of the studies were conducted in the Republic of Korea as parallel‐arm RCTs. The study subjects were overweight or obese with BMI of 23 kg/m^2^ or higher. Onion peel extract (OPE) was used in four studies as the IP, and one of the representative bioactive compounds was quercetin. Doses ranged from 100 to 468.4 mg/day for OPE and 28.6 to 100 mg/day for quercetin (Choi et al., 2015; Choi et al., 2020; Kim & Yim, 2015, 2016; Lee et al., 2016). In a study by Jeong et al. (2020), steamed onion capsule (900 mg/day) was used as the IP. The intervention period was 12 weeks in all studies, and the main evaluation markers were anthropometric parameters such as BW, BMI, BF, and WC. In addition, blood lipids, obesity‐related hormones, antioxidants, and endothelial function were measured.

**TABLE 1:** Characteristics of included studies.

First author (year)/location	Study design	Subjects' characteristics	Sample size	Types of supplementation	Period (weeks)	Evaluation markers
Choi et al. (2015)/Republic of Korea	RCT, Parallel	Overweight to obese – BMI >23 kg/m^2^	62 – Test, *n* = 36 – Placebo, *n* = 28	Onion peel extract 228 mg (100 mg quercetin) or placebo	12	Anthropometry – BW, BMI Biochemistry – TC, TG, LDL‐C, HDL‐C, glucose Endothelial function – FMD, EPCs
Choi et al. (2020) /Republic of Korea	RCT, Parallel	Overweight to obese – BMI >23 kg/m^2^	61 – Test, *n* = 31 – Placebo, *n* = 30	Onion peel extract 468.4 mg (100 mg quercetin) or placebo	12	Anthropometry – BW, BMI, BFM, %BF, FFM, ARFATP, LEFATP, TRFATP, AndFATP, FATP
Jeong et al. (2020)/Republic of Korea	RCT, Parallel	Obese – BMI 25–30 kg/m^2^	56 – Test, *n* = 28 – Placebo, *n* = 28	Steamed onion capsules 900 mg or placebo	12	Anthropometry – BW, BMI, WC, HC, WHR, %BF, BFM, LBM CT – Abdominal fat area (visceral, subcutaneous) Biochemistry – TC, TG, LDL‐C, HDL‐C Hormones – Adiponectin, leptin, T3, T4, TSH
Kim and Yim (2015, 2016)/Republic of Korea	RCT, Parallel	Overweight to obese – BMI >23 kg/m^2^	37 – Test, *n* = 18 – Placebo, *n* = 19	Onion peel extract 100 mg (28.6 quercetin) or placebo	12	Anthropometry – BW, BMI, WC, HC Biochemistry – TC, TG, LDL‐C, HDL‐C Oxidative stress – ROS, SOD
Lee et al. (2016)/Republic of Korea	RCT, Parallel	Overweight to obese – BMI >23 kg/m^2^	72 – Test, *n* = 36 – Placebo, *n* = 36	Onion peel extract 340 mg (100 mg quercetin) or placebo	12	Anthropometry – BW, BMI, WC, HC, Tricep, ARFAT, LEFAT, TRFAT, FATP, FAT, BP Biochemistry – TC, TG, LDL‐C, HDL‐C, glucose, insulin Hormones – leptin

Abbreviations: 8‐iso‐PGEF2α, 8‐iso‐prostaglandin F2α; ACE, angiotensin‐converting enzyme; AI, augmentation index; ALP, alkaline phosphatase; ALT, alanine aminotransferase; AMDA, asymmetric dimethylarginine; AndFATP, android fat percentage; Apo‐AI, apolipoprotein‐I; Apo‐B, apolipoprotein‐B; ARFATP, arm fat percentage; AST, aspartate aminotransferase; BFM, body fat mass; BMI, body mass index; BP, blood pressure; BW, body weight; CT, computerized tomography; EPCs, endothelial progenitor cells; FATP, total fat percentage; FFM, fat free mass; FMD, flow‐mediated dilation; HbA1c, hemoglobin A1c; HC, hip circumference; HDL‐C, high‐density lipoprotein cholesterol; HR, heart rate; hs‐CRP, high‐sensitivity C‐reactive protein; ICAM‐1, intercellular adhesion molecule 1; IsoP, isoprostanes; LBM, lean body mass; LDL‐C, low‐density lipoprotein cholesterol; LEFATP, leg fat percentage; oxLDL, oxidized low lipoprotein; %BF, percent body fat; RCT, randomized controlled trial; RHI, reactive hyperemia index; ROS, reactive oxygen species; SOD, superoxide dismutase; T3, triiodothyronine; T4, thyroxine; TC, total cholesterol; TG, triglyceride; TRFATP, trunk fat percentage; TSH, thyroid‐stimulating hormone; VCAM‐1, vascular cell adhesion molecule 1; WC, waist circumference; WHR, waist‐to‐hip ratio.

### 3.3 Risk of bias assessment

**FIGURE 2:**
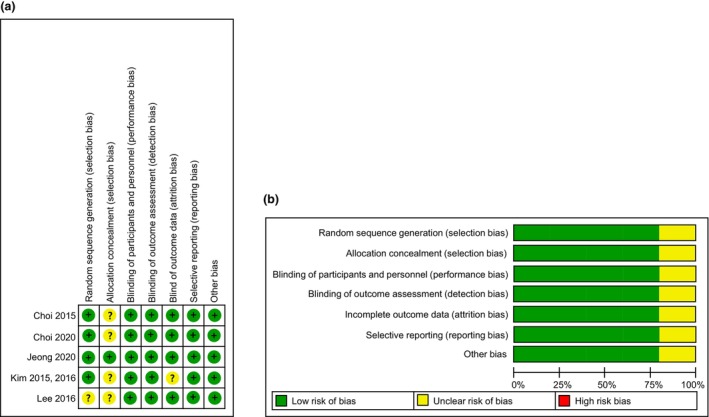
Assessment of risk of bias: (a) risk of bias summary: each risk of bias item for each included study, (b) risk of bias: each risk of bias item presented as percentages across all included studies.

The risk of bias in individual studies was evaluated by Cochrane's risk of bias tool (Figure 2). According to the criteria, more than 80% of the risk of bias was evaluated as low for all items except selection bias for allocation concealment. All studies were reported to have been randomized and double‐blind, but most of them were judged to be unclear with the exception of one study because there were no specific mentions of the method of allocation concealment. Performance bias, detection bias, reporting bias, and other biases were appropriately assessed in all studies and reported, and were judged to be low risk.

### 3.4 Antiobesity effects of onions

In 147 subjects from five studies (six articles), the effect of reducing BW and BMI by onion supplementation was quantified (Choi et al., 2015; Choi et al., 2020; Jeong et al., 2020; Kim & Yim, 2015, 2016; Lee et al., 2016). %BF and blood lipids were measured in 113 subjects from four studies (Choi et al., 2020; Jeong et al., 2020; Kim & Yim, 2015, 2016; Lee et al., 2016), and WC was measured in 82 subjects from three studies (Jeong et al., 2020; Kim & Yim, 2015, 2016; Lee et al., 2016). As a result of *I* ^2^ testing, TC and LDL‐C were judged to have high heterogeneity (*I* ^2^ = 59%, *I* ^2^ = 65%), and all other items were judged to have low heterogeneity (*I* ^2^ = 0%, *I* ^2^ = 0%, *I* ^2^ = 0%, *I* ^2^ = 22%, *I* ^2^ = 23%, *I* ^2^ = 0%, *I* ^2^ = 17%, *I* ^2^ = 0% for BW, BMI, %BF, WC, TG, HDL‐C, adiponectin, leptin). Therefore, TC and LDL‐C were analyzed with a random‐effects model, and other items were analyzed using a fixed‐effects model. In addition, in this meta‐analysis, there were only three to five data points per item in the selected studies, so publication bias could not be assessed due to a lack of statistical power (Dalton et al., 2016).

**FIGURE 3:**
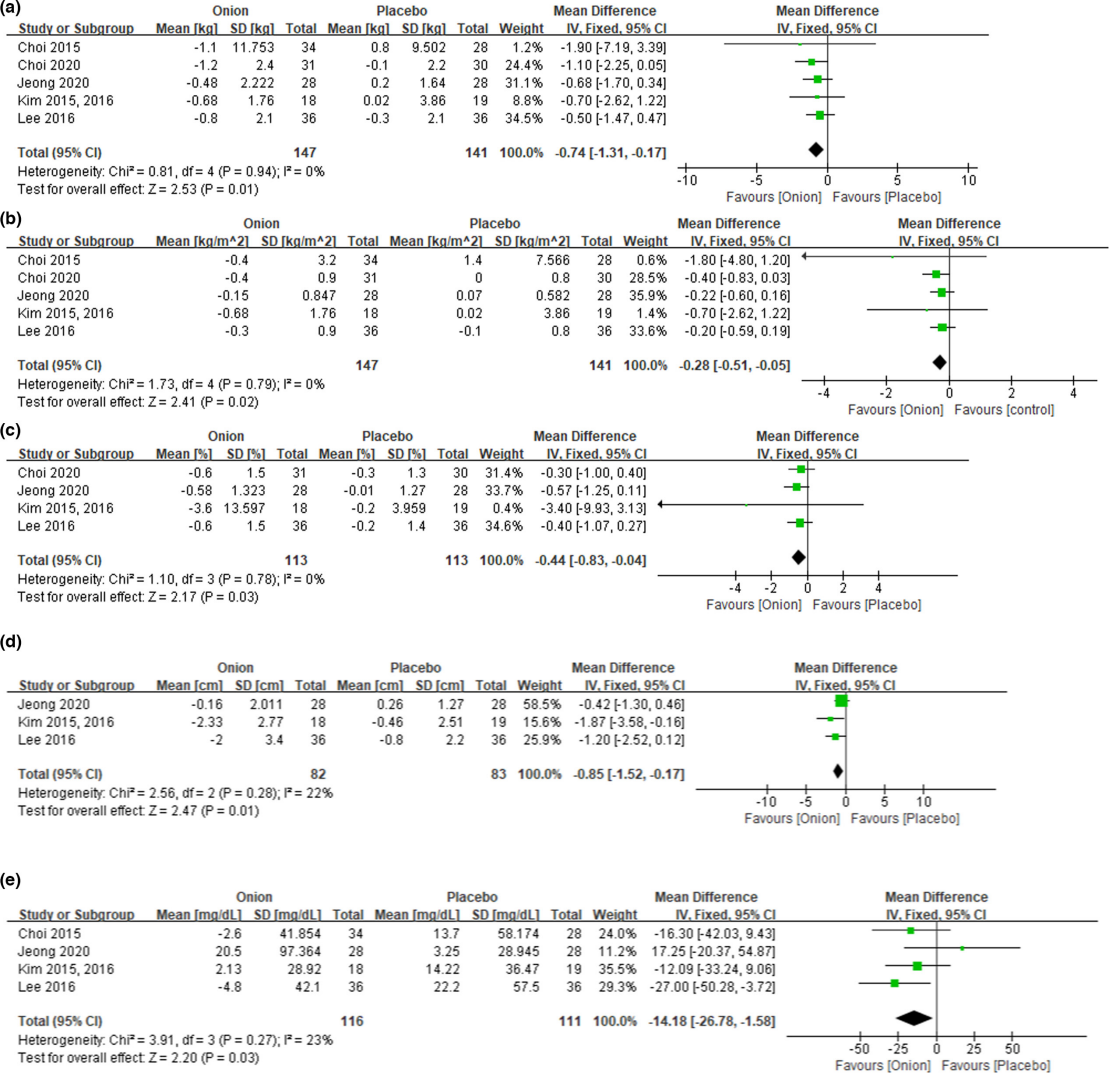
Forest plot for the effects of onions on obesity‐related parameters: (a) body weight, (b) body mass index, (c) percent body fat, (d) waist circumference, and (e) blood triglyceride levels.

After analyzing the changes in anthropometric parameters by onion supplementation, BW [weighted mean difference (WMD): 0.74 kg, 95% confidence interval (CI): −1.31 to −0.17], BMI (WMD: 0.28 kg/m^2^, 95% CI: −0.51 to −0.05), %BF (WMD: 0.44%, 95% CI: −0.83 to −0.04), and WC (WMD: 0.85 cm, 95% CI: −1.52 to −0.17) were statistically significantly decreased compared to placebo [*p* = .01, *p* = .02, *p* = .03, and *p* = .01, respectively; Figure 3a–d]. For blood lipid levels, triglycerides (WMD: 14.18 mg/dL, 95% CI: −26.78 to −1.58) were significantly reduced compared to placebo (*p* = .03; Figure 3e). In addition, there were no statistically significant differences in TC, HDL‐C, LDL‐C, or the obesity‐related hormones adiponectin and leptin (data not shown).

### 3.5 Sensitivity analysis

**FIGURE 4:**
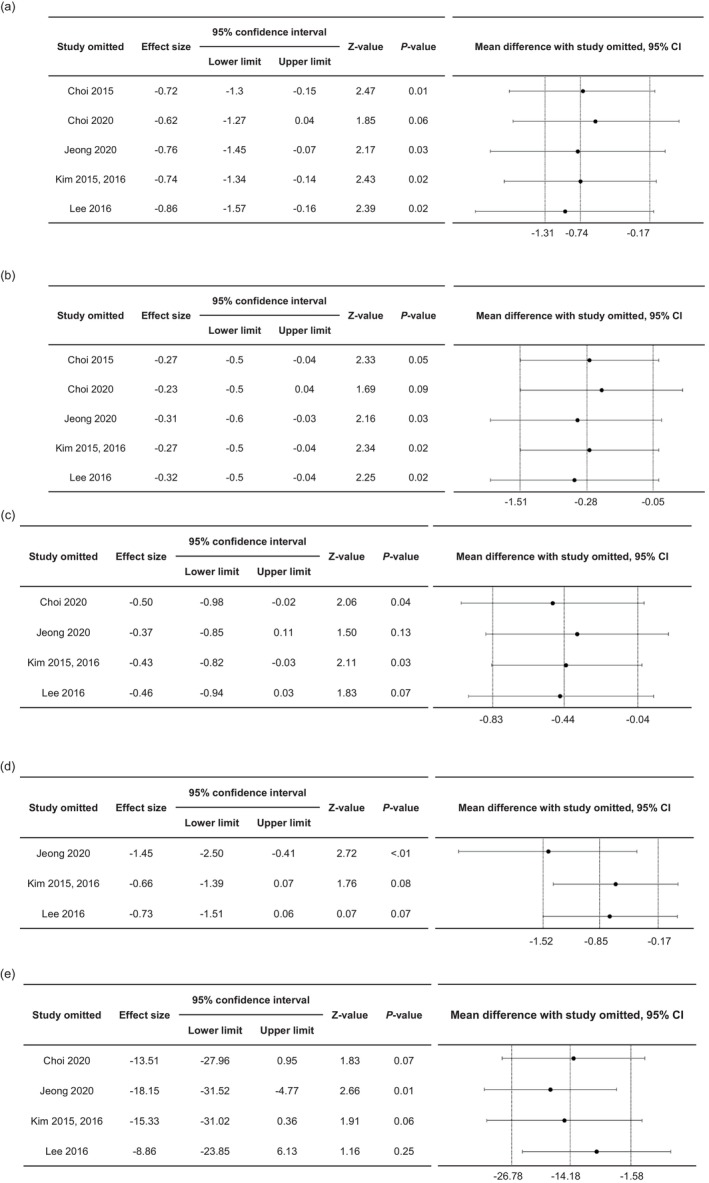
Sensitivity analysis: (a) body weight, (b) body mass index, (c) percent body fat, (d) waist circumference, and (e) blood triglyceride levels.

Sensitivity analysis was conducted using the leave‐one‐out method. The results revealed that BW (WMD: −0.62 kg, 95% CI: −1.27 to 0.04) and BMI (WMD: −0.23 kg/m^2^, 95% CI: −0.5 to 0.04) were influenced by Choi et al. (2015), %BF by Jeong et al. (2020) (WMD: −0.37%, 95% CI: −0.85 to 0.11) and Lee et al. (2016) (WMD: −0.46%, 95% CI: −0.94 to 0.03), WC by Kim & Yim (2015, 2016) (WMD: −0.66 cm, 95% CI: −1.39 to 0.07) and Lee et al. (27087901) (WMD: −0.73, 95% CI: −1.51 to 0.06), and TG by Choi et al. (2020) (WMD: −13.51 mg/dL, 95% CI: −27.96 to 0.95) and Lee et al. (2016) (WMD: −8.86 mg/dL, 95% CI: −23.85 to 6.13), which showed nonsignificant results (Figure 4a–e). When the study of Jeong et al. (2020) with different characteristics of raw materials was omitted, there was no change in the trend or statistical significance of the effect for all items except for %BF. Therefore, we determined that the effect of the IP type on antiobesity outcomes was low.

## 4 DISCUSSION

As interest in obesity management continues to grow, there is an increasing demand for dietary supplements that are safe and effective for weight loss. Among the various food ingredients and byproducts, onions contain an abundance of bioactive compounds and are known to be effective in various models of chronic metabolic disease (Babu & Srinivasan, 1997; Ebrahimi‐Mamaghani et al., 2014; Emamat et al., 2015; Mahmoud et al., 2021). Obesity is considered to be the main cause of chronic metabolic diseases, and we hypothesized that onion intake could elicit beneficial antiobesity effects. Therefore, we conducted an exhaustive review and meta‐analysis of clinical trials investigating the effects of onion on obesity in overweight or obese subjects.

To select studies for review, publications in the literature were searched using keywords including obesity, onion, and placebo. Following the review, five studies were selected from six articles. The selected studies were RCTs that were considered to have a low risk of bias. To evaluate the antiobesity effect of onion, data for anthropometric parameters were extracted from each study. BW and BMI were measured in all five studies, with three reporting a statistically significant decrease in the onion‐supplemented group (Choi et al., 2015; Choi et al., 2020; Lee et al., 2016). Although there were studies that did not show a significant decrease since the direction of decrease was consistent in these studies, a statistically significant decrease compared to placebo group could be confirmed by pooled data analysis (Jeong et al., 2020; Kim & Yim, 2015, 2016). %BF was measured in four studies. Since all of them reported a reduction effect (Choi et al., 2020; Jeong et al., 2020; Kim & Yim, 2015, 2016; Lee et al., 2016), a significant reduction effect can be confirmed in the results of pooled data analysis. WC was measured in three studies, with two reporting a reduction effect in the onion‐supplemented group (Kim & Yim, 2015, 2016; Lee et al., 2016). There was also a study that did not show a significant decrease in WC but since the direction of decrease was consistent (Jeong et al., 2020), a statistically significant reduction compared to placebo group could be confirmed by pooled data analysis. These effects of onion have been found to be modulated through various weight‐regulating mechanisms. The primary suspect is in the modulating effect of adipokines. Administration of onion peel extracts effectively reduces body fat by increasing adiponectin and decreasing leptin in a high‐fat diet (HFD)‐induced obesity model (Forney et al., 2018; Kim et al., 2012; Matsunaga et al., 2014). This effect is accompanied by the promotion of fatty acid oxidation. It was reported that administration of onion extract promotes fatty acid oxidation by increasing the expression of brown adipose tissue (BAT)‐related genes in 3T3‐L1 preadipocytes and adipocytes in HFD‐induced obese mice (Forney et al., 2018; Lee et al., 2017). The expression of these BAT‐related genes was regulated by the AMPK signaling pathway (Lee et al., 2017). The AMPK signaling pathway is also known to be involved in the regulation of lipid metabolism and alleviation of insulin resistance (Garcia & Shaw, 2017). Accordingly, several studies have reported that onion administration reduces blood lipid concentrations, fat mass accumulation, and insulin resistance (Desjardins & Steinberg, 2018; Henagan et al., 2015; Matsunaga et al., 2014; Yang et al., 2018; Yoshinari et al., 2012). Similar mechanisms have been identified with garlic, which belongs to the same genus *Allium* (Jayarathne et al., 2017), with garlic exhibiting antiobesity effects in several clinical trials (Choudhary et al., 2018; Sangouni et al., 2020).

Onion extract has also been shown to reduce lipid accumulation by inhibiting adipogenesis and fatty acid synthase activity (Wang et al., 2012; Yoshinari et al., 2012). In addition, onions were found to be effective in suppressing the inflammatory response accompanying obesity by reducing the expression of pro‐inflammatory markers such as CD68, MCP‐1, and PPARγ in adipose tissue (Forney et al., 2018; Kim et al., 2012). In this study, the lipid‐lowering effect of onion was also analyzed, and there was a statistically significant reduction in TG compared to the placebo group. Individually, three studies measured blood TG, of which only two studies with supplemented onion peels reported a decrease in levels. However, in the study by Jeong et al., which used peeled onion as a main ingredient, blood TG tended to increase (Jeong et al., 2020). From this, it can be speculated that the key component that reduces blood TG is the onion peel. Onion peels are known to contain high levels of quercetin, a type of flavonoid (Devarshi et al., 2017; Forney et al., 2018; Henagan et al., 2015; Kim & Kim, 2006). Quercetin has been attracting attention as a substance eliciting a preventive effect on chronic metabolic diseases due to its high antioxidant activity as well as inhibition of lipid accumulation, lipid metabolism regulation, and anti‐inflammatory effects (Boots et al., 2008; Hosseini et al., 2021). However, unlike the reported preclinical studies, there were no significant changes in other blood lipid parameters except for TG, so this should be interpreted with caution. In addition, since only trials conducted on obese subjects were selected for this study, additional analysis targeting subjects with dyslipidemia is warranted to verify the effect on blood lipids.

In summary, our study is the first systematic review and meta‐analysis to comprehensively analyze clinical trials focusing on the antiobesity effects of onions that have been conducted to date. We found convincing evidence that onions are effective not only in reducing body weight and body fat but also in reducing blood triglyceride levels. Although there have only been five robust clinical studies on the antiobesity effect of onions, and all of them have been conducted in Korea, these effects are generally in agreement on a consistent effect, with this effect being particularly pronounced in onion peels with high quercetin content.

## 5 CONCLUSIONS

In conclusion, the literature supports the finding that onion supplementation can significantly reduce body weight, body fat, and blood triglyceride levels. The effects were particularly noticeable with onion peels (a material that is often discarded during food processing), which may have applications as an adjunct therapy for weight control.

## AUTHOR CONTRIBUTIONS

Conceptualization: Chung MY and Park SH; Methodology: Chung MY, Hwang JT, and Park SH; Formal analysis: Chung MY and Park SH; Investigation: Chung MY, Hwang JT, and Park SH; Writing‐original draft: Chung MY and Park SH; Review and editing: Chung MY, Hwang JT, and Park SH.

## FUNDING INFORMATION

This study was funded by a research grant from the Korea Food Research Institute (Project number: E0210601).

## CONFLICT OF INTEREST STATEMENT

All contributing authors declare no conflicts of interest exist.

## Data Availability

Data sharing is not applicable to this article as no new data were created or analyzed in this study.

## References

[fsn33426-bib-0001] Alshaker, H. , Sacco, K. , Alfraidi, A. , Muhammad, A. , Winkler, M. , & Pchejetski, D. (2015). Leptin signalling, obesity and prostate cancer: Molecular and clinical perspective on the old dilemma. Oncotarget, 6(34), 35556–35563. 26376613 10.18632/oncotarget.5574 PMC4742124

[fsn33426-bib-0002] Babu, P. S. , & Srinivasan, K. (1997). Influence of dietary capsaicin and onion on the metabolic abnormalities associated with streptozotocin induced diabetes mellitus. Molecular and Cellular Biochemistry, 175(1–2), 49–57. 9350033 10.1023/a:1006881027166

[fsn33426-bib-0003] Barone, I. , Giordano, C. , Bonofiglio, D. , Ando, S. , & Catalano, S. (2016). Leptin, obesity and breast cancer: Progress to understanding the molecular connections. Current Opinion in Pharmacology, 31, 83–89. 27816025 10.1016/j.coph.2016.10.003

[fsn33426-bib-0004] Bastien, M. , Poirier, P. , Lemieux, I. , & Despres, J. P. (2014). Overview of epidemiology and contribution of obesity to cardiovascular disease. Progress in Cardiovascular Diseases, 56(4), 369–381. 10.1016/j.pcad.2013.10.016 24438728

[fsn33426-bib-0005] Boots, A. W. , Haenen, G. R. , & Bast, A. (2008). Health effects of quercetin: From antioxidant to nutraceutical. European Journal of Pharmacology, 585(2–3), 325–337. 18417116 10.1016/j.ejphar.2008.03.008

[fsn33426-bib-0006] Choi, E. Y. , Lee, H. , Woo, J. S. , Jang, H. H. , Hwang, S. J. , Kim, H. S. , Kim, W. , Kim, Y. S. , Choue, R. , Cha, Y. J. , Yim, J. E. , & Kim, W. (2015). Effect of onion peel extract on endothelial function and endothelial progenitor cells in overweight and obese individuals. Nutrition, 31(9), 1131–1135. 26233871 10.1016/j.nut.2015.04.020

[fsn33426-bib-0007] Choi, H. N. , Choue, R. , Park, Y. , & Yim, J. E. (2020). Onion Peel extract increases erythrocyte membrane n‐3 fatty acids in overweight and obese Korean subjects. Journal of Medicinal Food, 23(1), 37–42. 31855493 10.1089/jmf.2018.4366

[fsn33426-bib-0008] Choudhary, P. R. , Jani, R. D. , & Sharma, M. S. (2018). Effect of raw crushed garlic (*Allium sativum* L.) on components of metabolic syndrome. Journal of Dietary Supplements, 15(4), 499–506. 28956671 10.1080/19390211.2017.1358233

[fsn33426-bib-0009] Dalton, J. E. , Bolen, S. D. , & Mascha, E. J. (2016). Publication bias: The elephant in the review. Anesthesia and Analgesia, 123(4), 812–813. 27636569 10.1213/ANE.0000000000001596 PMC5482177

[fsn33426-bib-0010] Desjardins, E. M. , & Steinberg, G. R. (2018). Emerging role of AMPK in Brown and Beige adipose tissue (BAT): Implications for obesity, insulin resistance, and type 2 diabetes. Current Diabetes Reports, 18(10), 80. 30120579 10.1007/s11892-018-1049-6

[fsn33426-bib-0011] Devarshi, P. P. , Jones, A. D. , Taylor, E. M. , Stefanska, B. , & Henagan, T. M. (2017). Quercetin and quercetin‐rich red onion extract Alter Pgc‐1alpha promoter methylation and splice variant expression. PPAR Research, 2017, 3235693. 28191013 10.1155/2017/3235693 PMC5278221

[fsn33426-bib-0012] Ebrahimi‐Mamaghani, M. , Saghafi‐Asl, M. , Pirouzpanah, S. , & Asghari‐Jafarabadi, M. (2014). Effects of raw red onion consumption on metabolic features in overweight or obese women with polycystic ovary syndrome: A randomized controlled clinical trial. The Journal of Obstetrics and Gynaecology Research, 40(4), 1067–1076. 24612081 10.1111/jog.12311

[fsn33426-bib-0013] Emamat, H. , Foroughi, F. , Eini‐Zinab, H. , Taghizadeh, M. , Rismanchi, M. , & Hekmatdoost, A. (2015). The effects of onion consumption on treatment of metabolic, histologic, and inflammatory features of nonalcoholic fatty liver disease. Journal of Diabetes and Metabolic Disorders, 15, 25. 27453880 10.1186/s40200-016-0248-4 PMC4957858

[fsn33426-bib-0014] Forney, L. A. , Lenard, N. R. , Stewart, L. K. , & Henagan, T. M. (2018). Dietary quercetin attenuates adipose tissue expansion and inflammation and alters adipocyte morphology in a tissue‐specific manner. International Journal of Molecular Sciences, 19(3), 895–907. 29562620 10.3390/ijms19030895 PMC5877756

[fsn33426-bib-0015] Franssen, R. , Monajemi, H. , Stroes, E. S. , & Kastelein, J. J. (2011). Obesity and dyslipidemia. The Medical Clinics of North America, 95(5), 893–902. 21855698 10.1016/j.mcna.2011.06.003

[fsn33426-bib-0016] Garcia, D. , & Shaw, R. J. (2017). AMPK: Mechanisms of cellular energy sensing and restoration of metabolic balance. Molecular Cell, 66(6), 789–800. 28622524 10.1016/j.molcel.2017.05.032 PMC5553560

[fsn33426-bib-0017] Henagan, T. M. , Cefalu, W. T. , Ribnicky, D. M. , Noland, R. C. , Dunville, K. , Campbell, W. W. , Stewart, L. K. , Forney, L. A. , Gettys, T. W. , Chang, J. S. , & Morrison, C. D. (2015). In vivo effects of dietary quercetin and quercetin‐rich red onion extract on skeletal muscle mitochondria, metabolism, and insulin sensitivity. Genes & Nutrition, 10(1), 451. 25542303 10.1007/s12263-014-0451-1 PMC4277553

[fsn33426-bib-0018] Hosseini, A. , Razavi, B. M. , Banach, M. , & Hosseinzadeh, H. (2021). Quercetin and metabolic syndrome: A review. Phytotherapy Research, 35(10), 5352–5364. 34101925 10.1002/ptr.7144

[fsn33426-bib-0019] Jayarathne, S. , Koboziev, I. , Park, O. H. , Oldewage‐Theron, W. , Shen, C. L. , & Moustaid‐Moussa, N. (2017). Anti‐inflammatory and anti‐obesity properties of food bioactive components: Effects on adipose tissue. Preventive Nutrition and Food Science, 22(4), 251–262. 29333376 10.3746/pnf.2017.22.4.251 PMC5758087

[fsn33426-bib-0020] Jeong, S. , Chae, J. , Lee, G. , Shin, G. , Kwon, Y. I. , Oh, J. B. , Shin, D. Y. , & Lee, J. H. (2020). Effect of steamed onion (ONIRO) consumption on body fat and metabolic profiles in overweight subjects: A 12‐week randomized, double‐blind, placebo‐controlled clinical trial. Journal of the American College of Nutrition, 39(3), 206–215. 31368861 10.1080/07315724.2019.1635052

[fsn33426-bib-0021] Kagawa, Y. , Ozaki‐Masuzawa, Y. , Hosono, T. , & Seki, T. (2020). Garlic oil suppresses high‐fat diet induced obesity in rats through the upregulation of UCP‐1 and the enhancement of energy expenditure. Experimental and Therapeutic Medicine, 19(2), 1536–1540. 32010335 10.3892/etm.2019.8386 PMC6966189

[fsn33426-bib-0022] Kim, H. J. , Lim, S. , Chung, S. , Lee, S. , Choi, E. , Yang, K. H. , & Chung, M. Y. (2022). Barley sprout water extract and Saponarin mitigate triacylglycerol accumulation in 3T3‐L1 adipocytes. Journal of Medicinal Food, 25(1), 79–88. 35029509 10.1089/jmf.2021.K.0092

[fsn33426-bib-0023] Kim, K. A. , & Yim, J. E. (2015). Antioxidative activity of onion Peel extract in obese women: A randomized, double‐blind, placebo controlled study. Journal of Cancer Prevention, 20(3), 202–207. 26473159 10.15430/JCP.2015.20.3.202 PMC4597809

[fsn33426-bib-0024] Kim, K. A. , & Yim, J. E. (2016). The effect of onion Peel extract on inflammatory mediators in Korean overweight and obese women. Clinical Nutrition Research, 5(4), 261–269. 27812515 10.7762/cnr.2016.5.4.261 PMC5093223

[fsn33426-bib-0025] Kim, O. Y. , Lee, S. M. , Do, H. , Moon, J. , Lee, K. H. , Cha, Y. J. , & Shin, M. J. (2012). Influence of quercetin‐rich onion peel extracts on adipokine expression in the visceral adipose tissue of rats. Phytotherapy Research, 26(3), 432–437. 21833991 10.1002/ptr.3570

[fsn33426-bib-0026] Kim, S. J. , & Kim, G. H. (2006). Quantification of quercetin in different parts of onion and its DPPHRadical scavenging and antibacterial activity. Food Science and Biotechnology, 15(1), 39–43.

[fsn33426-bib-0027] Lanzotti, V. (2006). The analysis of onion and garlic. Journal of Chromatography. A, 1112(1–2), 3–22. 16388813 10.1016/j.chroma.2005.12.016

[fsn33426-bib-0028] Lee, J. S. , Cha, Y. J. , Lee, K. H. , & Yim, J. E. (2016). Onion peel extract reduces the percentage of body fat in overweight and obese subjects: A 12‐week, randomized, double‐blind, placebo‐controlled study. Nutrition Research and Practice, 10(2), 175–181. 27087901 10.4162/nrp.2016.10.2.175 PMC4819128

[fsn33426-bib-0029] Lee, S. G. , Parks, J. S. , & Kang, H. W. (2017). Quercetin, a functional compound of onion peel, remodels white adipocytes to brown‐like adipocytes. The Journal of Nutritional Biochemistry, 42, 62–71. 28131896 10.1016/j.jnutbio.2016.12.018

[fsn33426-bib-0030] Mahmoud, K. , Hammouda, A. Z. , Ali, H. , & Amin, A. (2021). Efficiency of red onion Peel extract capsules on obesity and blood sugar. Pakistan Journal of Biological Sciences, 24(1), 99–111. 33683036 10.3923/pjbs.2021.99.111

[fsn33426-bib-0031] Maierean, S. M. , Serban, M. C. , Sahebkar, A. , Ursoniu, S. , Serban, A. , Penson, P. , Banach, M. , & the Lipid and Blood Pressure Meta‐analysis Collaboration (LBPMC) Group . (2017). The effects of cinnamon supplementation on blood lipid concentrations: A systematic review and meta‐analysis. Journal of Clinical Lipidology, 11(6), 1393–1406. 28887086 10.1016/j.jacl.2017.08.004

[fsn33426-bib-0032] Marrelli, M. , Amodeo, V. , Statti, G. , & Conforti, F. (2018). Biological properties and bioactive components of *Allium cepa* L.: Focus on potential benefits in the treatment of obesity and related comorbidities. Molecules, 24(1), 119–136. 30598012 10.3390/molecules24010119 PMC6337254

[fsn33426-bib-0033] Matsunaga, S. , Azuma, K. , Watanabe, M. , Tsuka, T. , Imagawa, T. , Osaki, T. , & Okamoto, Y. (2014). Onion peel tea ameliorates obesity and affects blood parameters in a mouse model of high‐fat‐diet‐induced obesity. Experimental and Therapeutic Medicine, 7(2), 379–382. 24396409 10.3892/etm.2013.1433 PMC3881067

[fsn33426-bib-0034] Nishimura, M. , Muro, T. , Kobori, M. , & Nishihira, J. (2019). Effect of daily ingestion of quercetin‐rich onion powder for 12 weeks on visceral fat: A randomised, double‐blind, placebo‐controlled, parallel‐group study. Nutrients, 12(1), 91–102. 31905615 10.3390/nu12010091 PMC7019606

[fsn33426-bib-0035] Ren, F. , Reilly, K. , Kerry, J. P. , Gaffney, M. , Hossain, M. , & Rai, D. K. (2017). Higher antioxidant activity, Total Flavonols, and specific quercetin glucosides in two different onion (*Allium cepa* L.) varieties grown under organic production: Results from a 6‐year field study. Journal of Agricultural and Food Chemistry, 65(25), 5122–5132. 28612608 10.1021/acs.jafc.7b01352

[fsn33426-bib-0036] Saghafi‐Asl, M. , & Ebrahimi‐Mameghani, M. (2014). The effects of raw red onion consumption on serum levels of adiponectin, leptin, and hs‐CRP in overweight/obese females with polycystic ovarian syndrome: A randomized controlled‐clinical trial. Iranian Red Crescent Medical Journal, 19(11), e58674–e58681.

[fsn33426-bib-0037] Sangouni, A. A. , Mohammad Hosseini Azar, M. R. , & Alizadeh, M. (2020). Effects of garlic powder supplementation on insulin resistance, oxidative stress, and body composition in patients with non‐alcoholic fatty liver disease: A randomized controlled clinical trial. Complementary Therapies in Medicine, 51, 102428. 32507439 10.1016/j.ctim.2020.102428

[fsn33426-bib-0038] Seo, S. H. , Fang, F. , & Kang, I. (2021). Ginger (*Zingiber officinale*) attenuates obesity and adipose tissue remodeling in high‐fat diet‐fed C57BL/6 mice. International Journal of Environmental Research and Public Health, 18(2), 631–643. 33451038 10.3390/ijerph18020631 PMC7828532

[fsn33426-bib-0039] Trisat, K. , Wong‐on, M. , Lapphanichayakool, P. , Tiyaboonchai, W. , & Limpeanchob, N. (2017). Vegetable juices and fibers reduce lipid digestion or absorption by inhibiting pancreatic lipase, cholesterol solubility and bile acid binding. International Journal of Vegetable Science, 23(3), 260–269.

[fsn33426-bib-0040] Uzunlulu, M. , Telci Caklili, O. , & Oguz, A. (2016). Association between metabolic syndrome and cancer. Annals of Nutrition & Metabolism, 68(3), 173–179. 26895247 10.1159/000443743

[fsn33426-bib-0041] Wang, Y. , Tian, W. X. , & Ma, X. F. (2012). Inhibitory effects of onion (*Allium cepa* L.) extract on proliferation of cancer cells and adipocytes via inhibiting fatty acid synthase. Asian Pacific Journal of Cancer Prevention, 13(11), 5573–5579. 23317220 10.7314/apjcp.2012.13.11.5573

[fsn33426-bib-0042] Yang, C. , Li, L. , Yang, L. , Lu, H. , Wang, S. , & Sun, G. (2018). Anti‐obesity and Hypolipidemic effects of garlic oil and onion oil in rats fed a high‐fat diet. Nutrition & Metabolism (London), 15, 43. 10.1186/s12986-018-0275-x PMC6011244 29951108

[fsn33426-bib-0043] Yoshinari, O. , Shiojima, Y. , & Igarashi, K. (2012). Anti‐obesity effects of onion extract in Zucker diabetic fatty rats. Nutrients, 4(10), 1518–1526. 23201769 10.3390/nu4101518 PMC3497009

[fsn33426-bib-0044] Zhao, X. X. , Lin, F. J. , Li, H. , Li, H. B. , Wu, D. T. , Geng, F. , Ma, W. , Wang, Y. , Miao, B. H. , & Gan, R. Y. (2021). Recent advances in bioactive compounds, health functions, and safety concerns of onion (*Allium cepa* L.). Frontiers in Nutrition, 8, 669805. 34368207 10.3389/fnut.2021.669805 PMC8339303

[fsn33426-bib-0045] Zhou, D. D. , Mao, Q. Q. , Li, B. Y. , Saimaiti, A. , Huang, S. Y. , Xiong, R. G. , Shang, A. , Luo, M. , Li, H. Y. , Gan, R. Y. , Li, H. B. , & Li, S. (2022). Effects of different green teas on obesity and non‐alcoholic fatty liver disease induced by a high‐fat diet in mice. Frontiers in Nutrition, 9, 929210. 35811941 10.3389/fnut.2022.929210 PMC9263825

